# Location of Hyperintense Vessels on FLAIR Associated with the Location of Perfusion Deficits in PWI

**DOI:** 10.3390/jcm12041554

**Published:** 2023-02-16

**Authors:** Lisa D. Bunker, Argye E. Hillis

**Affiliations:** 1 Department of Neurology, Johns Hopkins University School of Medicine, Baltimore, MD 21287, USA; 2 Departments of Cognitive Science, Physical Medicine & Rehabilitation, and Neurology, Johns Hopkins University School of Medicine, Baltimore, MD 21287, USA

**Keywords:** acute stroke, hypoperfusion, perfusion-weighted imaging (PWI), fluid-attenuated inversion recovery (FLAIR), hyperintense vessels

## Abstract

Perfusion imaging is preferred for identifying hypoperfusion in the management of acute ischemic stroke, but it is not always feasible/available. An alternative method for quantifying hypoperfusion, using FLAIR-hyperintense vessels (FHVs) in various vascular regions, has been proposed, with evidence of a statistical relationship with perfusion-weighted imaging (PWI) deficits and behavior. However, additional validation is needed to confirm that areas of suspected hypoperfusion (per the location of FHVs) correspond to the location of perfusion deficits in PWI. We examined the association between the location of FHVs and perfusion deficits in PWI in 101 individuals with acute ischemic stroke, prior to the receipt of reperfusion therapies. FHVs and PWI lesions were scored as present/absent in six vascular regions (i.e., the ACA, PCA, and (four sub-regions of) the MCA territories). Chi-square analyses showed a significant relationship between the two imaging techniques for five vascular regions (the relationship in the ACA territory was underpowered). These results suggest that for most areas of the brain, the general location of FHVs corresponds to hypoperfusion in those same vascular territories in PWI. In conjunction with prior work, results support the use of estimating the amount and location of hypoperfusion using FLAIR imaging when perfusion imaging is not available.

## 1. Introduction

In acute ischemic stroke, current clinical standards in diagnosis and intervention planning include perfusion-weighted MRI or CT. These diagnostic tools can be used in various ways (e.g., to identify the core volume or a mismatch ratio across various imaging sequences) to rapidly identify if there is hypoperfused tissue that may benefit from reperfusion interventions, such as intravenous tissue-type plasminogen activator (IV tPA) or mechanical thrombectomy (MT). Furthermore, they can be useful to corroborate a neurological exam, or for research, to investigate brain–behavior relationships, and identify the areas of dysfunction (hypoperfusion and/or infarct) associated with specific deficits in acute stroke, before the opportunity for reorganization or recovery [1,2,3]. However, perfusion-weighted imaging may not be feasible because of contraindication for contrast agents [4,5,6] or other patient factors, or may not be useful due to technical difficulties. Arterial spin labelling (ASL) sequences are another option for identifying perfusion deficits, but ASL is technically complicated and sensitive to motion artifact [7,8,9], and thus may be difficult to acquire and interpret.

**Figure 1:**
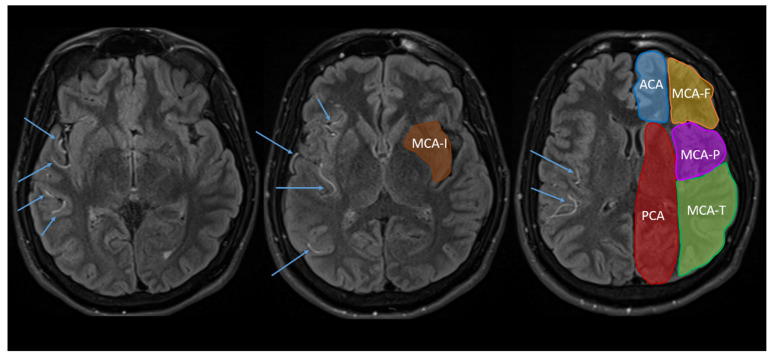
Examples of FHVs (indicated by the arrows) and vascular regions used for the NIH-FHV score: the anterior cerebral artery (ACA, blue), posterior cerebral artery (PCA, red), and four regions of the middle cerebral artery (MCA)—the MCA-frontal (MCA-F, orange), MCA-temporal (MCA-T, green), MCA-parietal (MCA-P, purple), and MCA-insular (MCA-I, brown). Note that no FLAIR imaging for the current study was shared by the NIH. Thus, this example of FHVs is from a participant in Bunker et al. [13].

Reyes and colleagues proposed an alternative method for identifying hypoperfusion using fluid-attenuated inversion recovery (FLAIR) MRI sequences [10], which are routinely collected in stroke MRI protocols. Perfusion abnormalities on FLAIR present as a hyperintense signal in the arterial vessels, which appears as serpentine structures and/or isolated bright spots in the sulci (see Figure 1). FLAIR-hyperintense vessels (FHVs) are indicative of reduced blood flow [11,12], and thus may serve as an alternative avenue for the identification and quantification of hypoperfusion. Indeed, Reyes et al. developed the National Institutes of Health FHV (NIH-FHV) score to quantify the number and location of FHVs (in terms of the anterior cerebral artery (ACA), posterior cerebral artery (PCA), and four regions of the middle cerebral artery (MCA) territory). They demonstrated a strong significant association between the NIH-FHV scores and PWI lesion volume (i.e., one point on the NIH-FHV scale equated to about 12 mL of hypoperfusion on PWI). When controlling for lesion volume on diffusion-weighted imaging, the NIH-FVH was highly sensitive at detecting a mismatch ratio ≥ 1.8. However, Reyes and colleagues did not examine whether the location of FHVs corresponded to the location of the hypoperfusion in PWI.

To aid in the clinical interpretation of FHVs, it is necessary to establish a relationship between the location(s) of FHVs compared to the regions of the hypoperfusion identified using dynamic contrast PWI. That is, do FHVs in a specific vascular area actually correspond to a perfusion deficit in that same area, as seen in PWI? Without such validation, the NIH-FHV scale could not be reliably used in clinical practice or research. In a previous smaller study (*n* = 73), we found statistically significant relationships for FHV/PWI lesions in each vascular area, except the ACA territory [13], presumably due to low power (only three participants demonstrated FHVs in the ACA territory). The purpose of the current investigation was to examine the association between the location of FHVs on FLAIR and hypoperfusion in PWI in a larger cohort (i.e., the same cohort studied by Reyes et al.), to hopefully replicate our previous findings, and potentially identify an association for the ACA territory. The identification of significant relationships in the location of the hypoperfusion identified on the different MRI sequences will improve the clinical and research utility of the NIH-FHV score when PWI is not available.

## 2. Materials and Methods

PWI and FHV rating data are from the National Institutes of Health (NIH) Natural History of Stroke study (for protocol, see NCT00009243, https://www.clinicaltrials.gov). Enrollment and data collection (described in Reyes et al. [10]) was approved by the NIH institutional review board (IRB) and all participants or legally authorized representatives provided informed consent as per the Declaration of Helsinki. The current analysis, using de-identified data provided by the NIH, was approved by the Johns Hopkins Medicine IRB.

### 2.1. Participants

A total of 101 participants (53 male sex, 48 female sex; median (range) age = 73 (58–83)) with hyperacute unilateral (right or left) ischemic stroke were included in this analysis. They all completed an MRI with diffusion-weighted imaging (DWI), FLAIR and PWI, prior to receiving IV tPA or MT interventions. See Table 1 for additional sample characteristics (such as stroke severity, lesion volume, premorbid hypertension, hyperlipidemia, smoking history, etc.).

### 2.2. Imaging Acquisition

MRIs were acquired using 1.5T (GE Signa Scanner, General Electric Medical Systems) or 3T (Philips Achieva, Philips Healthcare; Siemens Skyra, Siemens AG) scanners. Parameters for FLAIR acquisition were: repetition time (TR), 9000 ms; echo time (TE), 120–145 ms; 3.5 mm slice thickness (40 slices). Dynamic susceptibility contrast PWI parameters were: TR, 1–1.5 s; TE, 25–45 ms; 7 mm slice thickness (20 slices with a full brain coverage); 40–80 dynamics. For PWI, participants received 0.1 mmol/kg of gadolinium–diethylenetriamine penta-acetic acid (Magnevist, Bayer Schering Pharma) or gadobenic acid MultiHance (Bracco Diagnostics) contrast at 5 mL/s flow rate.

### 2.3. Perfusion Deficit Identification

#### 2.3.1. FLAIR-Hyperintense Vessels

For the initial NIH-FHV scoring, FLAIR images were reviewed slice by slice and the presence of FHVs was scored from 0 to 2 in each of the six vascular regions in the lesioned hemisphere. A score of ‘0’ indicated no FHVs, a score of ‘1’ indicated 1–2 FHVs on 1–2 slices, and a score of ‘2’ indicated 3+ FHVs on one slice or 3+ slices with FHVs. Scores for each region were summed for a total score out of 12 for the hemisphere, with higher scores suggestive of a greater volumes of hypoperfusion. The six vascular regions (see Figure 1) were the ACA territory, the PCA territory, and four sub-regions of the MCA territory (frontal (i.e., MCA-F), temporal (i.e., MCA-T), parietal (i.e., MCA-P), and insular (i.e., MCA-I)). Since the purpose of this study was simply to identify the association between the location of hypoperfusion across FLAIR and PWI MR techniques, these scores were recoded as a binary variable, indicating that FHVs—or hypoperfusion—were/was present or absent in each region. Hence, a score of 1–2 in any of the six regions was recoded as 1, for ‘present’, or 0, for ‘absent.’ The relationship between total FHV scores (i.e., severity of hypoperfusion) and PWI volumes are reported elsewhere [10].

#### 2.3.2. PWI Volume

**Figure 2:**
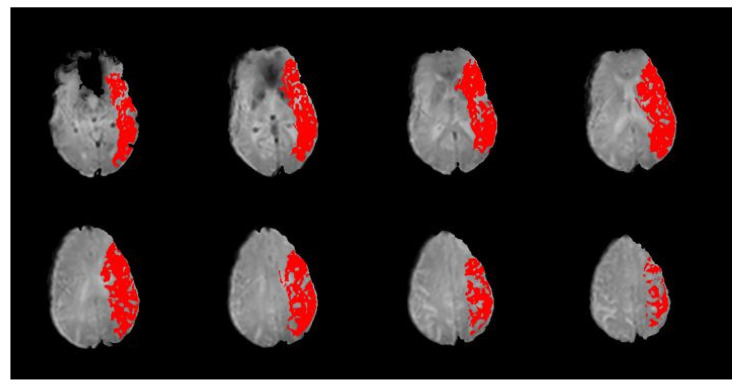
Sample slices from a participant’s PWI scan with time-to-peak (TTP) map overlay. A TTP delay of ≥4 s is shown in red. Per the slices included here, this participant would be coded as having a perfusion deficit present in all four regions of the MCA territory (frontal (MCA-F), temporal (MCA-T), parietal (MCA-P), and insular (MCA-I)).

Time-to-peak (TTP) maps were created from PWI sequences by subtracting the TTP (i.e., time until maximal contrast concentration) for each voxel of normal tissue in the contralesional hemisphere from corresponding voxels in the ipsilesional hemisphere. A perfusion deficit was defined as a greater than 4-s delay in the TTP. TTP maps were created by an NIH investigator, and then de-identified before being shared for this study. An example is provided in Figure 2. For this analysis, while blinded to the NIH-FHV score, perfusion deficit was coded as present or absent in each of the six vascular regions as described previously (viz., ACA, PCA, MCA-F, MCA-T, MCA-P, and MCA-I).

An association between the presence/absence of FHVs and hypoperfusion in PWI was calculated for each vascular region using a Pearson’s chi-square (*Χ*^2^), with the strength of association (comparable to Pearson’s *r*) calculated using Cramér’s *V*.

## 3. Results

Lesion metrics, such as DWI lesion volume and volume of hypoperfusion in PWI, and a median/range of NIH-FHV scores for the sample are reported in Table 1. With regards to the binary FHV score, 78% (*n* = 78) of the participants had FHVs in at least one vascular region, with 3% (*n* = 3) with FHVs in the ACA territory, 8% (*n* = 8) in the PCA territory, 54% (*n* = 55) in the MCA-F, 58% (*n* = 59) in the MCA-T, 33% (*n* = 33) in the MCA-P, and 60% (*n* = 61) in the MCA-I. Seventy-nine percent (*n* = 80) of the sample demonstrated perfusion deficits in PWI, with 5% (*n* = 5) of the sample showing hypoperfusion in the ACA territory, 12% (12) in the PCA territory, 40% (*n* = 40) in the MCA-F, 48% (*n* = 48) in the MCA-T, 60% (*n* = 61) in the MCA-P, and 37% in the MCA-I (*n* = 37). As NIH-FHV scores for this sample were completed by another lab, we also examined the agreement between binary FHV and PWI variables as an indication of an agreement across labs. There was a 92% point-to-point agreement on the presence/absence of hypoperfusion on both MRI sequences (i.e., scored as having FHVs and perfusion deficit in PWI, or the opposite: no FHVs and no PWI deficit) with 71% of the sample demonstrating both FHVs and perfusion deficit in PWI.

Results of the chi-square analysis (see Table 2) show a statistically significant association between the presence of FHVs and hypoperfusion in PWI for each vascular region, although the relationship in the ACA territory did not survive Bonferroni’s correction (i.e., *p* < 0.008). That is, for ACA, *Χ*^2^ = 5.29, *p* = 0.02; for PCA, *Χ*^2^ = 12.06, *p* = 0.001, for MCA-F, *Χ*^2^ = 33.74, *p* < 0.000; for MCA-T, *Χ*^2^ = 36.58, *p* < 0.000; for MCA-P, *Χ*^2^ = 15.48, *p* < 0.000; and for MCA-I, *Χ*^2^ = 28.55, *p* < 0.000. For all analyses, df = 1 and *n* = 101. Per Cramer’s *V*, the relationship between the presence of FHVs and perfusion deficit in PWI in the ACA territory is ‘strong’ (i.e., *V* > 0.15), with all other vascular regions having ‘very strong’ associations (i.e., *V* > 0.25) [14] between the two approaches. Because there were so few participants demonstrating FHVs or perfusion deficits in PWI in the ACA territory, we subsequently conducted a Fisher’s exact test for this region, but it was also non-significant (Fisher’s exact = 0.143).

## 4. Discussion

### 4.1. Clinical Importance of Hypoperfusion

#### 4.1.1. Stroke Diagnosis and Localization

The pathophysiology of cerebrovascular ischemia varies for tissue where the blood flow is completely restricted compared to tissue where the blood flow is reduced (i.e., hypoperfused, or the ischemic penumbra), but both can cause deficits in neurological function [15]. A key difference, however, is the potential to restore perfusion and subsequent neurologic functions to the penumbra. In fact, the ischemic penumbra is the target for all acute stroke interventions [16]. Often, endovascular treatment is based on the volume of the penumbra, but the location of the penumbra should be considered in weighing the potential risks and benefits of intervention. To illustrate, reperfusion of the non-infarcted left temporal gyrus is likely to restore the ability to understand language [17] and to produce meaningful speech, which may be considered a potential benefit that would outweigh substantial risks. On the other hand, reperfusion of the right superior temporal gyrus would be likely to restore more subtle cortical functions, such as understanding the emotional tone of voice [18], recognizing facial expressions [19,20], and empathy [21]. While these functions are important for social interactions, the potential benefit of restoring these functions might not be weighed favorably against the risks of intervention in cases where the intervention carries the risk of hemorrhagic conversion of a relatively large infarct (outside the penumbra). Likewise, reperfusion of the right occipital cortex would be likely to restore the left visual field, which would be especially important for individuals who depend on full vision, such as pilots and truck drivers. Identification of the location of the penumbra is therefore essential for clinicians to provide valuable information to individuals with stroke and their caregivers, to allow a fully informed consent for treatment on the basis of predicted risks and benefits. Our findings will assist clinicians in providing this information based on the location of hypoperfusion, even when perfusion imaging is not available (because of allergy to contrast, inadequate IV access, or lack of necessary technology).

In fact, the window for reperfusion treatment has been expanded well beyond the initial 4–6 h in cases where a penumbra can be demonstrated. The benefit of endovascular treatment has been demonstrated up to 24 h (e.g., DAWN trial, [22]); the benefit of reperfusion with temporary blood pressure elevation to reperfuse the penumbra has been demonstrated up to 1 week after the onset of symptoms in cases of large vessel stenosis and large penumbra [23]; and the benefit of encephaloduroarteriosynangiosis, where there is penumbra in the presence of the moya-moya syndrome weeks after the onset of stroke [24]. However, in these cases, it is also essential for the individual to fully understand the potential benefits in terms of functions that can be restored, in order to make informed decisions about treatment.

Thus, the need to identify and differentiate penumbra from infarct has become a critical need in acute stroke management, both to determine appropriate interventions, and to assess the response to those interventions [15,25]. Identification of hypoperfused tissue on imaging is also important in differential diagnosis through the confirmation of localization of symptoms seen on neurological exam. While perfusion imaging is a core component of the current guidelines for the management of acute ischemic stroke [26], here we have shown, in conjunction with other research [27,28,29], that FLAIR MRI is another useful tool for the diagnosis and management of (hyper) acute ischemic stroke, and is also much more routinely collected. With the discovery of hyperintense vessels, and their association with abnormal hemodynamic function [11,12,30], FLAIR imaging has become an alternative tool for identifying hypoperfusion when PWI is not an appropriate or successful option. Reyes and colleagues [10] demonstrated a novel use for FLAIR imaging to identify and quantify perfusion deficits as a proxy for PWI. However, additional investigation for this new tool was needed to support its clinical application during stroke diagnosis and care management. That is, validation of the location of FHVs relative to the perfusion deficits in PWI was needed.

In our prior work, we showed a significant relationship between the location of FHVs and perfusion deficits in PWI [13], but that study was in a retrospective analysis of imaging data from a subset of participants in a prospective, longitudinal study examining stroke recovery in right and left hemisphere strokes. Additionally, because the study was retrospective, some participants’ FLAIR imaging was acquired under different specifications (i.e., with different slice thicknesses), so some participants had more opportunities to demonstrate FHVs than others. Although we reported significant associations with regards to localization in five of six vascular regions, the limitations warranted additional investigation to validate the NIH-FHV tool. We subsequently aimed to replicate our findings by analyzing this larger sample of prospectively collected data.

#### 4.1.2. Investigation of Neuroanatomical Function and Organization

Many investigations of neurological (dys) function, utilizing brain lesions, have been conducted in individuals in chronic stages of recovery, after reorganization has likely occurred. Thus, examinations of lesion–deficit relationships would ideally be conducted in premorbidly healthy individuals as soon as possible following injury onset, such as in an acute stroke. Since behavioral presentation associated with neural dysfunction corresponds not only to regions of frank lesion, but regions of hypoperfused tissue as well, it is helpful and appropriate for researchers to incorporate measures of hypoperfusion when undertaking investigations in acute/early subacute populations, before reperfusion therapy [3,31,32,33]. Furthermore, the strongest evidence that an area of the brain is critical to a function is obtained when the function is impaired when the neural region is hypoperfused, and recovers when the neural region is reperfused [32]. Although better methods of measuring hypoperfusion may be available (i.e., PWI or ASL), there are various reasons why an investigator may want to adopt the NIH-FHV scale utilizing FLAIR imaging, such as cost, safety (with regards to administering contrast), and accessibility. While Reyes et al. [10] showed a strong association between the estimated amount of hypoperfusion on FLAIR and the actual volume of hypoperfusion identified in PWI, they did not investigate any associations in location. Without clear evidence that the location on one corresponded to the location on the other, the findings of brain–behavior relationships, in terms of localization of function, using the NIH-FHV might be, at worst, erroneous, or, at best, speculative.

This analysis demonstrates that for five of the six vascular regions—as defined by the NIH-FHV—the general location of FHVs does in fact significantly correspond with general areas of PWI deficits of ≥4 s TTP delays. Unfortunately, we failed to identify a significant association between FHV and PWI lesion in the ACA territory (after correcting for multiple comparisons). As with our prior study, we suspect this was due to having too few participants with FHVs or PWI lesions in the ACA territory for statistical comparison (i.e., only *n* = 3 with FHVs and *n* = 5 with PWI deficits). This finding was unsurprising given the fact that ischemic stroke due to ACA vessel occlusion is relatively uncommon (i.e., less than 2% of ischemic strokes per some studies) [34,35]. Thus, additional investigation is still needed to determine if there is a relationship between the two MRI methods in this specific vascular territory.

With validation of the location of hypoperfusion in these regions, the current study offers some validation of our previous work examining associations between NIH-FHV and behavioral outcomes [13,36]. For example, we reported a functional relationship between FHV scores in the MCA frontal region and performance on the NIH Stroke Scale (NIHSS) [37], which has been shown to be biased towards anterior lesions [38] and/or motor and language modalities [39,40]. NIHSS scores were collected for this sample, but per our data-sharing agreement with the NIH, we did not have access to this data in order to examine any association between NIH-FHV scores and NIHSS scores, so we are unable to replicate this finding with the current sample.

We have also demonstrated a relationship between NIH-FHV scores in the MCA parietal region, and content production during a discourse task and picture naming, independent of the lesion volume and age/education [13]. In another study, we found that various tests of hemispatial neglect were associated with NIH-FHV scores in different regions (e.g., NIH-FHV score in the MCA-P region was independently associated with line bisection, while viewer- and stimulus-centered errors on a gap detection task were associated with scores in the MCA-T and MCA-F areas, respectively) [36]. Although these various findings were statistically significant, the full impact of the results depended on more clear evidence that the location of FHVs does indeed correspond with hypoperfusion in those same regions, which this study provides. Again, we were not provided with any individual behavioral data for this sample, if it was collected, so we could not examine any relationships between NIH-FHV and behavior as part of this analysis. Regardless, our prior studies do show the utility of using the NIH-FHV score to examine brain–behavior relationships beyond the clinical examination.

### 4.2. Conclusions and Future Directions

These results, in conjunction with our previous study [13], indicate that FHVs in the MCA and PCA territories may be reliably associated with hypoperfusion in those same areas. Thus, not only can the NIH-FHV score be used to quantify the volume of hypoperfusion (see [10]), it can be used to identify the general location of the hypoperfusion in the MCA and PCA vascular territories as well (although additional investigation in the ACA territory is needed). Information about the location of the hypoperfusion allows the clinician to predict what functions can be restored with intervention to restore blood flow, which is information that is critical for informed decision making. These findings are important for confirming the localization of deficits seen on neurological exam. These results also validate the associations with behavioral performance we reported previously, such as the significant independent relationship between FHVs in the MCA-F region and NIHSS scores [13,36]. Since individual NIHSS scores—or other behavioral outcomes—for this data set were not available to us, we were unable to replicate our findings with regards to the behavioral performance in this cohort. Nonetheless, collectively, these studies support use of the NIH-FHV scale in both clinical and research settings. Although perfusion imaging would be the ideal method for identifying the location and volume of perfusion deficits, and likely available for many patients with concern for stroke, care providers and investigators may be able to identify the location and estimate the volume of hypoperfusion using FLAIR imaging. This tool may also allow investigators to examine brain–behavior relationships in acute/early subacute patients, before reorganization has occurred.

## Figures and Tables

**Table 1:** Participant characteristics.

Summary Statistics (*n* = 101)	
Age (Median (Range))	73 (58–83)
Sex (female, male)	48, 53
PWI volume (mL; *M(SD)*)	37 (±56)
DWI volume (mL; *M*(*SD)*) †	18 (±24)
NIH-FHV (median (range))	4 (1–6)
NIHSS (median (range))	8 (4–17)
Stroke Risk Factors	
HTN	*n* = 73 (72.3%)
HLD	*n* = 34 (33.7%)
DM	*n* = 20 (19.8%)
CAD	*n* = 15 (14.9%)
A-Fib	*n* = 31 (30.7%)
Smoking	*n* = 12 (11.9%)

Notes: PWI volume = volume of hypoperfusion on perfusion-weighted imaging; DWI = infarct volume on diffusion-weighted imaging; NIH-FHV = National Institutes of Health FLAIR-Hyperintense Vessel score; NIHSS = National Institutes of Health Stroke Scale; HTN = hypertension; HLD = hyperlipidemia; DM = diabetes; CAD = coronary artery disease; A-Fib = arterial fibrillation. † DWI lesion calculated from the number of voxels within the PWI lesion (after co-registration) below 620 µm^2^/s [10].

**Table 2:** Chi-Square results (presence of FHVs and hypoperfusion in PWI).

Vascular Region	Chi-Square (*Χ*^2^)	*p*-Value	Cramér’s *V*
ACA	5.29	0.02	0.23
PCA	12.06	0.001 *	0.35
MCA-F	33.74	0.000 *	0.58
MCA-T	36.58	0.000 *	0.60
MCA-P	15.48	0.000 *	0.39
MCA-I	28.55	0.000 *	0.53

Notes: df = 1, *n* = 101 for all *Χ*^2^ calculations; FHVs = FLAIR-hyperintense vessels; PWI = perfusion-weighted imaging; ACA = anterior cerebral artery; PCA = posterior cerebral artery; MCA = middle cerebral artery; F = frontal region of the MCA; T = temporal region of the MCA; P = parietal region of the MCA; I = insular region of the MCA. * statistically significant at *p* < 0.008.

## Data Availability

Quantitative data are available upon request, but restrictions apply to the data obtained from the NIH. It may be available from the authors with the permission of the NIH.
